# The relationships of the pulmonary arteries to lung lesions aid in differential diagnosis using computed tomography

**DOI:** 10.7603/s40681-015-0011-z

**Published:** 2015-06-09

**Authors:** Chien-Heng Lin, Tsai-Chung Li, Po-Pang Tsai, Wei-Ching Lin

**Affiliations:** 1 Department of Pediatrics, Children’s Hospital, China Medical University Hospital, 404 Taichung, Taiwan; 2 Department of Biomedical Imaging and Radiological Science, China Medical University, 404 Taichung, Taiwan; 3 Graduate Institute of Biostatistics, China Medical University, 404 Taichung, Taiwan; 4 Department of Radiology, China Medical University Hospital, 404 Taichung, Taiwan; 5 School of Medicine, China Medical University, 404 Taichung, Taiwan; 6 Department of Radiology, China Medical University Hospital, No. 2, Yuh-Der Road, 404 Taichung, Taiwan

**Keywords:** Lung lesion, Pulmonary artery, Multidetector computed tomography

## Abstract

The improvement of the resolution of rapid scanning in multidetector computed tomography (CT) has an increased accuracy that allows for the demonstration of the relationship of the pulmonary arteries and lung lesions, even in the peripheral lung. The purpose of this study is to evaluate the relationship between the pulmonary arteries and lung lesions by CT, and to use this relationship to distinguish between benign and malignant lung lesions. The relationships of the lung lesions and the adjacent pulmonary artery were recorded as encasement, displacement, penetration, in the margin, and disconnection. Statistical analyses were then performed to evaluate the relationship of the pulmonary arteries to each lesion with a focus toward the possibility of malignancy and the degree of pulmonary arterial encasement in the lesion. The relationship between the pulmonary arteries and lung lesions had a statistically significant difference between benignancy and malignancy (*P* < 0.001). Inter-observer agreement was substantial (κ = 0.639; 95% CI: 0.518-0.719). The average degrees of pulmonary arterial encasement in benign and malignant lesions were 52.1% ± 27.3% and 71.8% ± 18.8%, respectively (*P* = 0.011). The ROC curve showed that the degree of pulmonary arterial encasement had a moderate discriminating ability in diagnosing lung carcinoma, and the area under the curve was 0.738. The best cutoff value was 44.4%. The relationships of the pulmonary arteries to lung lesions and the degree of pulmonary arterial encasement could be used in differentiating benignancy from malignancy not only for central lung lesions but also peripheral lung lesions.

## 1. Introduction

A solitary pulmonary tumor is a common radiological finding. And while computed tomography (CT) is considered to be one of the most important non-invasive diagnostic tools in clinical practice, differentiating between a malignant and a benign lesion on CT is not an easy task for a radiologist [1, 2].

The lung has a dual blood supply: one source is the bronchial arteries, and the other is the pulmonary arteries. Generally, the bronchial artery provides the blood supply to bronchogenic carcinoma. Using the relationship of the bronchial artery to lung lesions in distinguishing said lesions’ benignancy from malignancy in CT usually has limitations because tracing the whole course of the bronchial artery to the lung carcinoma is still a challenge [3].

The pulmonary vessels are usually encased or invaded by lung carcinoma. Therefore, the relationship of nodules to both the pulmonary arteries and veins has been reported as the parameter in the neural network to distinguish benignancy from malignancy [4, 5].

In our preliminary study, we found that the pulmonary vein was easily encased, compressed, or invaded by benign and malignant lesions due to low blood pressure, but we were unable to differentiate whether each vein was encased, compressed, or invaded by each lesion.

The pulmonary artery has a relatively higher blood pressure than the pulmonary vein, and so may not be as easily affected as the pulmonary vein. Therefore, it may be better used as a parameter to differentiate benignancy from malignancy.

Improvement of the temporal and spatial resolution in the multidetector CT (MDCT), along with rapid coverage of the whole thorax and power auto-injector and CT software techniques, has made it possible to time the initiation of a scan to take into consideration peak enhancement so that the pulmonary arteries can be shown clearly, even in the peripheral lung. In addition, multiple windows and levels are visible simultaneously with multiplanar reformations (MPR), and maximum or minimum intensity projections (MIP and minIP) tools can be used interactively at the workstation so that radiologists can trace the whole course of each pulmonary artery and differentiate the relationships between the lung lesion more accurately than with conventional CT, even in the peripheral small lung nodules [1].

**Table 1:** Methods to prove the final diagnosis in malignancy and benignancy.

Methods to prove final diagnosis	Case numbers
**Malignancy**	77
Ultrasonography guided biopsy	10
CT guided biopsy	3
Transbronchial biopsy	41
Transbronchial brush	33
Transbronchial wash	33
Thoracotomy	14
**Benignancy**	23
CT guided biopsy	2
Transbronchial wash	4
Thoracotomy	4
Sputum culture and F/U	13

CT = computed tomography, F/U = follow up until the lesion resolves (about 6-18 months).

The aim of this study was to evaluate the ability of the relationships between the pulmonary arteries and lung lesions to distinguish benignancy from malignancy in said lesions. In addition, the degree of pulmonary arterial encasement in predicting malignancy was evaluated with the receiver operating characteristic (ROC) curve.

## 2. Patients and methods

### 2.1. Patients

We reviewed all of the chest CT images from August 2005 to December 2005. There were 100 patients with solitary pulmonary nodules or mass who underwent a 16-slice MDCT study. The final diagnosis was made by the pathological biopsy or the sputum culture (Table 1). Age and sex were assessed in patients with either benign or malignant lesions. This study was approved by the Institutional Review Board with patients signing a waiver of informed consent.

### 2.2. CT protocol and image analysis

All of the CT imaging was performed with a 16-slice MDCT (LightSpeed and BrightSpeed, General Electric Medical Systems). Before dynamic CT was performed, high resolution CT (HRCT) images were obtained. One-mm-thick images were taken at 10-mm spacings from lung apexes to lung bases, with the patient breathing out fully for each image. Then, 100 ml of iodinated contrast medium (Omnipaque 350 mg I/ml) was intravenously injected with a power injector at a rate of 3 ml/sec. The scanning was then performed from the lung apexes to the middle pole of both kidneys. Smart preparation technique-scanning delay was automatically determined with bolus tracking in the aorta, and only after the contrast medium injection and the subsequent waiting for the density of aorta to be over 120 Hu was the starting scan used. The region of interest (ROI) was placed in the aortic arch. The scanning parameters were a collimation of 1.25 mm, a table speed of 34.375 mm/sec and a pitch of 1.375. Axial slices were reconstructed with a slice width of 5.0 mm and a slice interval of 5.0 mm. Then, the 0.625 mm reconstructed raw data of the dynamic CT images were sent to the CT workstation (Advantage Window 4.4).

**Fig. 1:**
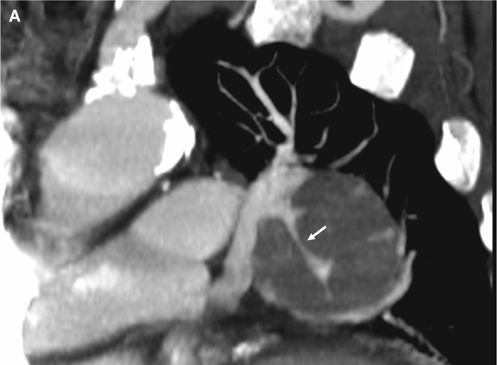

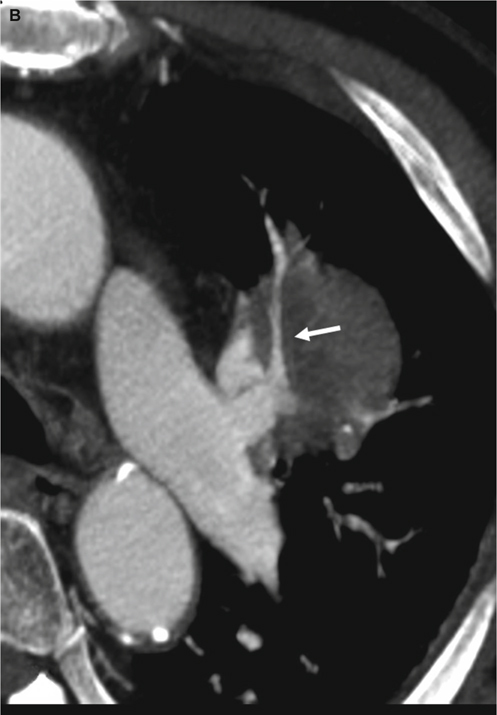
Encasement. This is an 82-year-old male with a mass lesion abutting the left hilum. He received bronchoscopic biopsy and pathology revealed small cell lung carcinoma. Coronal (A) and axial (B) section computed tomography (CT) images reveal the main tumor envelops the left segmental pulmonary artery, with decrease in the diameter of this artery (white arrows).

**Fig. 2:**
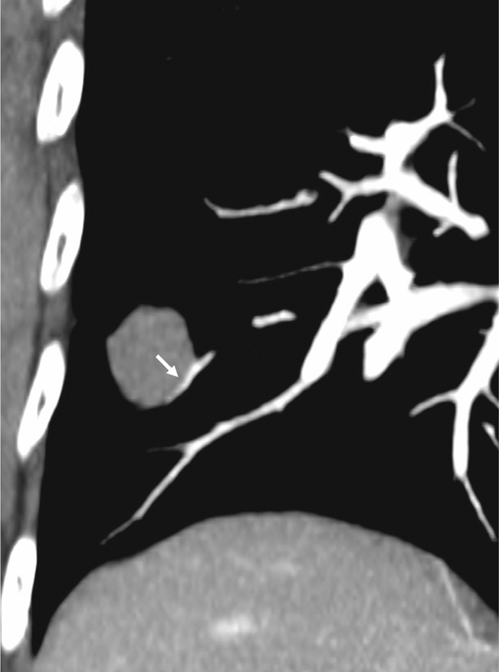
Displacement. This is a 68-year-old female with a pulmonary nodule in the right lower lobe. She received right lower lobe lobectomy, and pathology revealed adenocarcinoma. Coronal reformatted CT image reveals the nodule pushing the peripheral right pulmonary artery away from its normal vascular course with a smooth indentation on the pulmonary artery (white arrow).

Two radiologists with 15 years (Observer 1) and 3 years (Observer 2) of experience in chest CT independently reviewed the nearly-isotropic data set consisting of 300-400 reconstructed CT images at the workstation. They were both familiar with the CT workstation techniques and unaware of the final diagnosis of each lung lesion. Several tools were used at the workstation to display data, and, most often, MPR and MIP were used in our study. Rotations around and along any axis in real time were also used. The observers assessed the relationship of the lung lesions and adjacent pulmonary artery with multiple windows and levels simultaneously at CT workstations and recorded the relationships as encasement, displacement, penetration, in the margin, and disconnection.

**Fig. 3:**
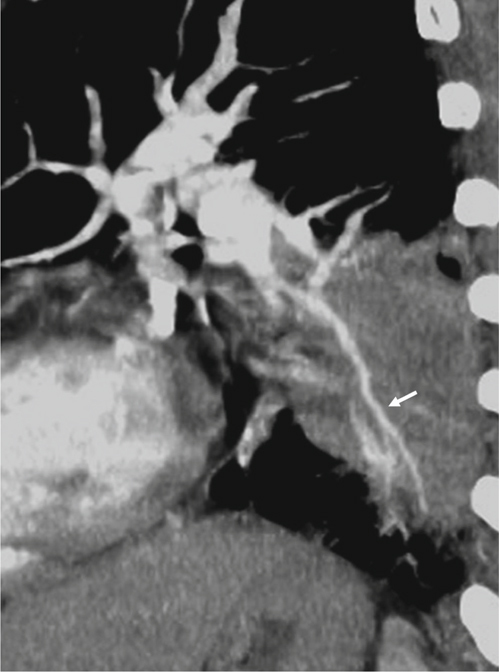
Penetration. This is a 64-year-old male with a mass lesion in the left lower lobe. He received ultrasonographyguided biopsy, and pathology revealed adenocarcinoma. Sagittal reformatted CT image reveals the pulmonary artery passes through the lesion without change of the vascular course or caliber of the pulmonary artery (white arrows).

The definition of encasement is a mass enveloping a pulmonary artery while decreasing the size of a pulmonary artery’s caliber (Figure 1). Displacement indicates a mass is causing deviation of a pulmonary artery away from the normal vascular course with/without a notch in the pulmonary artery (Figure 2). Penetration is when a pulmonary artery passes through the lesion without changing the vascular course or caliber of that pulmonary artery (Figure 3). In the margin is when a pulmonary artery passes across the lesion’s margin but without changing its vascular course or its caliber. Disconnection is when there is no pulmonary artery contact with the lesion. More than one type of relationships would be recorded if more than one pulmonary artery was in connection with the lesion. The degree of mass effect in order is encasement, displacement, and then penetration. The mass effect that cannot be clarified as a lesion is the mass effect that involves disconnection or in the margin to/with a pulmonary aftery, so these types of lesions were not included in the statistical analyses. Finally, the observers recorded the relationship of each pulmonary artery with the most mass effect of each lesion in the statistical analyses.

As well, two chest radiologists also measured the size of all of the lesions and recorded the location of each lesion as central or peripheral. A central lesion is a lesion that occurs in the hilar bronchus (main, lobar, or segmental bronchus), while a peripheral lesion is one that occurs below the level of the segmental bronchus.

When the result of the same nodule was not the same from the 2 observers, the results were discussed by both observer 1 and 2, and then they reached a consensus.

**Fig. 4:**
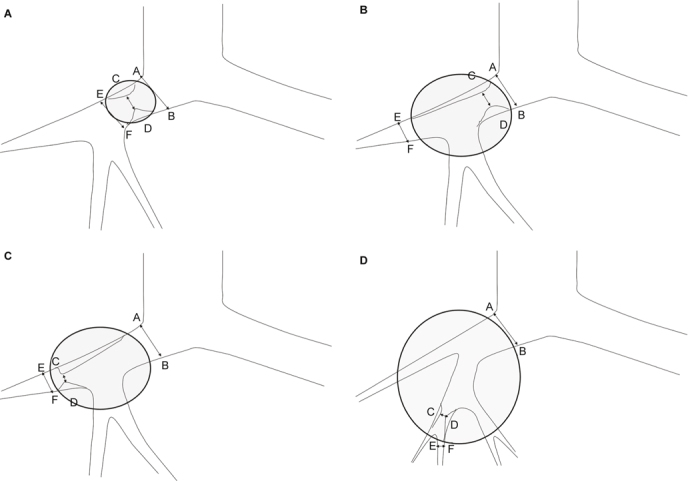
(A) A lesion (circle) encases the pulmonary artery, and the encased segment doesn’t include a bifurcation. The degree of encasement was determined by the ratio between the luminal diameter at the point of greatest stenosis, CD, and the normal artery beyond the lesion at proximal end, AB. The degree of encasement = (1-CD/AB)^*^100%; this was labeled as type A encasement. (B) A lesion (circle) encases the pulmonary artery, and the encased segment includes a bifurcation, the narrowest segment is proximal to the bifurcation. The degree of encasement = (1-CD/AB)^*^100%; this was labeled as type B encasement. (C) A lesion (circle) encases the pulmonary artery, and the encased segment includes a bifurcation, the narrowest segment is distal to the bifurcation. The degree of encasement = (1-CD/EF)^*^100%; EF was the normal artery beyond the lesion at distal end. This was labeled as type C encasement. (D) A lesion (circle) encases the pulmonary artery and the encased segment includes at least 2 bifurcations. The narrowest point is at the segment between 2 bifurcations. The degree of encasement = (1-CD/CD’)^*^100%, CD’= EF+EC/EA^*^(AB-EF); CD’ was an approximation of an expected normal diameter of the narrowest segment, EC was the distance between the narrowest point to the normal segment beyond the lesion at distal end. EA was the distance of the vessel at normal segments between both ends of the lesion.

In addition, as pulmonary arterial encasement was recorded, the degree of encasement was measured by the 2^nd^ observer at the workstation. When there were different degrees of encasement of a pulmonary artery or the lesion was encased by more than one pulmonary artery, he would repeatedly place the tracer in all of the narrowed segments by zooming in on the images, using different thicknesses of the MIP images, checking the tracer in all of the planes, and rotating around the tracer at the workstation. After repeat calculations, he recorded the data showing the most advanced degree of encasement. The degree of encasement was calculated and the methods are listed in Figure 4, categorized by how many pulmonary arterial bifurcations were within the lesion and the location of the narrowest segment of each pulmonary artery. When the vessel was encased, very lightly enhanced, and barely visualized, it was difficult to measure, so it was be recorded as amputation, and the degree of encasement was 100%.

### 2.3. Statistical analyses

Statistical analyses were performed with SAS software (version 9.13; SAS Institute, Cary, NC). Each patient’s gender and the relationship between lung lesion and adjacent pulmonary artery in the benign and malignant groups were analyzed by using Fisher’s exact test. Two-independent-sample *T*-test was used to analyze statistically significant difference in patients’ ages, and a Wilcoxon rank sum test was used to analyze the degree of pulmonary arterial encasement by each lung lesion in the benign and malignant groups. Odds ratio with its 95% confidence interval (CI) was used to estimate the strength of the association between malignancies and the relationships of the pulmonary arteries to the lung lesions by using the group of those who had lesions with pulmonary artery penetration as the baseline. A *P* value of less than 0.05 was considered to indicate a significant difference.

**Table 2:** The relationships of pulmonary arteries to the lung lesions recorded by 2 observers and the final results.

	Benignancy			Malignancy		
Relationships/Observers	1	2	Final results	1	2	Final results
Encasement	8	6	8	56	59	58
Displacement	2	1	2	28	25	27
Penetration	10	13	10	4	4	4
In the margin	0	0	0	1	1	1
Disconnection	3	3	3	2	2	2

**Table 3:** Basic data compared between benignancy and malignancy.

	Benignancy	Malignancy	*P* value
Age (years old)	57.2 ± 22	67 ± 11	*P* = 0.074
Gender (female:male)	6:14	24:50	*P* = 1
Location (C:P)	9:11	46:28	*P* = 0.13
Tumor size (mm) (mean ± SD)	51.1 ± 17.4	48.1 ± 21.9	*P* = 0.578

C = central located, P = peripheral lesion, SD = standard deviation.

**Table 4:** The relationships of pulmonary arteries to the lung lesions recorded by 2 observers and the final results.

	Benignancy			Malignancy		
Relationships/Observers	1	2	Final results	1	2	Final results
Encasement	4	8	8	56	59	58
Displacement	1	2	2	28	25	27
Penetration	15	10	10	4	4	4
In the margin	0	0	0	1	1	1
Disconnection	3	3	3	2	2	2

Inter-observer agreement was assessed by using kappa (κ) statistics, which measures the level of agreement after taking chance agreements into account. Furthermore, the sensitivity and specificity profiles of the degree of pulmonary arterial encasement in the diagnosis of malignancy were determined by plotting an empirical ROC curve.

## 3. Results

The relationships between pulmonary arteries and lung lesions were recorded by Observer 1 and Observer 2, and the final results are listed in Table 2. Six nodules (3 malignant and 3 benign lesions) were not included in the statistical analyses because in these cases the pulmonary arteries were in the margin or disconnect with the lung lesions. Finally, 74 patients who had bronchogenic carcinomas and 20 patients who had benign lung lesions were included in the statistical analyses. The final diagnoses of these patients are listed in Table 3. The basic data analyses are shown in Table 4.

There were 15 malignant lesions that had 2 different types of relationship to the pulmonary arteries. The relationship of the pulmonary arteries to the benign lesions and the malignant ones was statistically significantly different (*P* < 0.001). A lesion with a pulmonary arterial encasement had an 18.1 times greater likelihood of being malignant than a lesion with a pulmonary artery penetrating through it (95% CI: 4.6-71.7, *P* < 0.001). A lesion that displaced the pulmonary artery had a 33.7 times greater likelihood of being malignant than a lesion with a pulmonary artery penetrating through it (95% CI: 5.3-213.7, *P* = 0.0002).

Inter-observer agreement was substantial (κ = 0.612; 95% CI: 0.518-0.719).

**Fig. 5:**
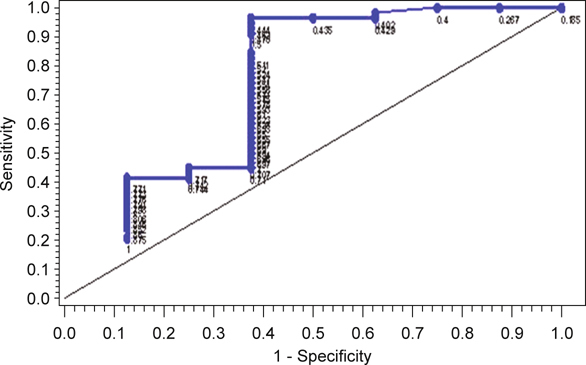
The ROC curve for diagnosis of malignancy using the degree of pulmonary arterial encasement. Points labeled in the picture are the values of degree of encasement.

The lesions with different types of pulmonary arterial encasement that were related to the calculation of the degree of encasement are presented in Table 5. The average degrees of pulmonary arterial encasement in benign and malignant lesions were 52.1% ± 27.3% and 71.8% ± 18.8% (*P* = 0.011), respectively. The empirical ROC curve for the diagnosis of a malignant lung tumor with the degree of pulmonary arterial encasement showed a moderate discriminating ability in diagnosing lung carcinoma, and the area under the ROC curve was 0.738 (Figure 5). The best cutoff value for the degree of pulmonary arterial encasement in the diagnosis of malignancy was 44.4%. If 44.4% was chosen as the cutoff value for diagnosing malignancy of a lung tumor with said degree of pulmonary arterial encasement, then the sensitivity was 96.6% (56/58), specificity was 62.5% (5/8), positive predictive value was 94.9% (56/59), and negative predictive value was 71.4% (5/7), respectively. The 2 false negative results came from squamous cell carcinoma, and these 2 nodules both had 40% encasement. The 3 false positive results came from 3 tuberculosis (TB) lesions with 71%, 74% and 100% encasement, respectively.

**Table 5:** The type of pulmonary artery encasement that related to the calculation of the degree of encasement

Types of encasement	Benignancy (case number)	Malignancy (case number)
Type A	0	0
Type B	0	10
Type C	1	5
Type D	6	32
Amputation	1	11

Type A = The encased segment didn’t include any bifurcation, as in Figure 4A; Type B = The encased segment included one bifurcation, and the narrowest point was located proximate to the bifurcation, as in Figure 4B; Type C = The encased segment included one bifurcation, and the narrowest point was located distal to the bifurcation, as in Figure 4C; Type D = The encased segment included more than one bifurcation, as in Figure 4D.

## 4. Discussion

CT is the most cost-effective image modality for evaluating lung lesions. A benign nodule can be diagnosed by either there being a benign pattern of calcification or the nodule having a doubling time of more than 2 years there being a benign pattern of calcification, by using fat, or by the nodule having a doubling time of more than 2 years [6, 7]. However, if the lesion does not have these features, the radiologist must use the morphologic signs from CT, like attenuation, margin, contours, cavitation, size, enhanced pattern and clinical information, to judge if the lesion is malignant [1, 2, 4, 5, 7-9]. But, there are several overlaps between benign and malignant lesions.

With the increased use of ultrasonography (US) to evaluate peripheral lung lesions, Hsu *et al*. found that a pulmonary artery vessel signal, a pulsatile flow with vessel signal length ≥ 1 cm demonstrated by color Doppler US, could be a sign for predicting benign lesions (10). However, this finding cannot be applied to CT scans, since the length of the pulmonary arteries within the lesions; both benign and malignant, is always seen in CT as more than 1 cm if the diameter of the lesion is more than 1 cm.

The blood supply for lung cancer is mainly from the bronchial arteries, and Tan *et al*. revealed that 16-slice MDCT demonstrated that fact by showing that bronchial arteries were the blood supply for 94% of lung cancers [3]. According to Muller’s report, the largest possible diameter of a bronchial artery supply to the bronchogenic carcinoma depends on tumor size. When the tumor is 1-2 cm, the largest lumen of the feeding bronchial artery is 100 µm, and when the tumor is 2-4 cm, the largest lumen of the feeding bronchial artery is 200-600 µm [11]. The minimal thickness of 16-slice MDCT is 0.625 mm, meaning that it may not be sufficient to demonstrate the whole vascular course of the feeding bronchial artery or for a tumor size of less than 4 cm or even bigger. The detected rate of blood supply for lung lesions was 42% (41 malignancies and one benign) in our study, which is lower than Tan’s report. This may be due to the smaller sizes and marginal locations of the lesions we studied. In addition, some bronchial arteries also supply several non-cancerous lung lesions [12], so we could not always use the feeding bronchial arteries to distinguish between benign and malignant lesions.

At an early stage of tumor development, the bronchial arteries have a regular hilifugal course to supply the tumor, and then a wool ball-like pattern at an advanced stage. When a tumor is larger than 4-6 cm in diameter, it will show regression and the necrotic area of the tumor will be noted. Then it may have a central avascular cavity and increased vascularization in the growth zone at its edges, with bronchial artery-to-pulmonary artery anastomoses [11]. The pulmonary arteries mainly supply the lung parenchyma and several alveolar-originated lesions, but sometimes they are found to supply bronchogenic carcinomas. The histological cell type, tumor grading, tumor size, tumor location, and grade of differentiation of bronchogenic carcinomas have been associated with the possibility of the pulmonary arteries supplying blood to lung carcinomas. Zhang YL *et al*. found that pulmonary arteries had a greater tendency to supply to a big, peripherally located, low-differentiated carcinoma and to supply to squamous cell carcinoma than to adenocarcinoma [13]. This may be due to the rapid growth of these types of tumor and the fact that it receives relatively less blood supply from the bronchial arteries in peripheral lung lesions than in central lung lesions. However, this is rarely evidenced in radiological images, possibly due to poor tissue contrast for bronchogenic carcinoma and lung parenchyma during pulmonary arterial angiography.

Computer-aided detection (CAD) and artificial neural networks have been developed to improve the detection rate and ability in differential diagnosis [4, 5, 9]. Their ability is dependent on the input data, and these systems are at present used as a second opinion to assist radiologists and are not routinely used in daily practice. It is a well-known fact that vascular encasement by a tumor is likely a sign of that tumor’s malignancy, such as pancreatic head carcinoma and hepatocellular carcinoma encasing a portal vein. Chen *et al*. used the relationship of the nodule to blood vessels as one of the nine CT signs of the neural networkbased CAD scheme in distinguishing a malignant from a benign solitary pulmonary nodule. They classified the relationships into 3 groups: disconnection with blood vessels, connection with blood vessels without notching, and connection with blood vessels with notching [4]. Matsuki *et al*. recorded the vascular involvement of the lung nodule from no involvement (score 0) to the involvements of more than two vessels (score 10). They used this as one of the 16 radiological findings and combined this with 7 clinical parameters to build up a neural network scheme for differentiating benign from malignant pulmonary nodules on HRCT [5]. Both studies suggest that these neural network-based CAD schemes could assist radiologists in improving the overall diagnostic accuracy in differentiating between benign and malignant tumors on HRCT.

With the improvement of the temporal and spatial resolution in MDCT due to dynamic protocol and several useful tools such as MPR, MIP and VR at the workstation, we can clearly trace the pulmonary arteries to the peripheral lung about 2 cm away from pleura. As our study shows, we can use the relationship between the pulmonary arteries and lung lesions to distinguish benignancy from malignancy, since the relationship of the pulmonary arteries to lung lesions has a significant difference between benignancy and malignancy. In short, we found that malignant lesions had a tendency to encase and displace the pulmonary arteries, and benign lesions had a tendency to be penetrated by the pulmonary arteries. Furthermore, when using 50% pulmonary arterial encasement as the cut-off value, the sensitivity and the specificity in predicting malignancy were 91.4% and 62.5%, respectively. This result is easy to apply in clinics and may further be used as a sign in CAD schemes.

The more desmoplastic reaction of inflammatory lesions, like TB, might show prominent mass effect and different histology cancer cell types, and grades of malignant cell differentiation may also express different degrees of mass effect on adjacent pulmonary arteries. These circumstances may cause some limitations in using the relationship between the vessel and the tumor and the degree of pulmonary artery encasement to differentiate benignancy from malignancy in lung lesions.

The quantification of observer agreement is an essential complement in conventional studies of diagnostic accuracy in the evaluation of a diagnostic test [14]. Agreement between these relationships in our study was also determined, using the κ statistic. The level of agreement was considered poor with κ < 0.20, fair with κ = 0.21-0.40, moderate with κ 0.41-0.60, substantial with κ = 0.61-0.80, and very good with κ > 0.80 [15]. The interobserver agreement was substantial (κ = 0.639), which was similar to the agreement found in previous studies of mammography and other CT interpretive tasks [16].

The limitation of this study is the small sample size of benign lesions. This may have lead to the sharp slope of the ROC curve, which, as the degree of encasement decreases from 70.8% to 44.4%, inversely increases the sensitivity from 44.8% to 96.6% with a fixed specificity of 62.5%. Also, this study didn’t include the lesions of metastasis and bronchoalveolar carcinoma, which were thought to have blood supplies mainly from the bronchial arteries. This suggests the possibility that there may be another story in the relationship of these lesions to the pulmonary arteries. Furthermore, the lack of benign tumors in our benign lesion group was also noted. Further investigation to include more variant origins of benign lesions and different histologic types of malignancy is needed.

Besides, the resolution and enhancement of the pulmonary arteries at 1-2 cm away from the pleura depends on several factors, such as ratio of the amount of contrast medium to patients’ body size and contrast medium injection flow rate. Because we have difficulty tracing the pulmonary arteries at this region with confidence, this may cause the routine application of our results to have limitations in evaluating these very peripherally located small lesions.

## 5. Conclusion

In this study, we have shown that the relationships of the pulmonary arteries to lung lesions and the degree of pulmonary arterial encasement could help in differentiating benignancy from malignancy in lesions, not only for central lung lesions, but also for peripheral lung lesions located below the 2^nd^-order bronchus to 2 cm away from pleura.

## Acknowledgments

There are no acknowledgments.

## Ethics committee approval

The Institutional Review Board approved this retrospective study with a waver of the written informed consent.

## Conflict of interest

The authors declare that they have no conflicts of interest.
